# Relationship between spine osteoarthritis, bone mineral density and bone turn over markers in post menopausal women

**DOI:** 10.1186/1472-6874-10-25

**Published:** 2010-08-08

**Authors:** Linda Ichchou, Fadoua Allali, Samira Rostom, Loubna Bennani, Ihsane Hmamouchi, Fatima Z Abourazzak, Hamza Khazzani, Laila El Mansouri, Redouane Abouqal, Najia Hajjaj-Hassouni

**Affiliations:** 1 Laboratory of Information and Research on Bone Diseases (LIRPOS). Department of Rheumatology, El Ayachi hospital, University Hospital of Rabat-Sale, Morocco; 2 Laboratory of Biostatistical, Clinical and Epidemiological Research (LBRCE). Faculty of Medicine and Pharmacy, Rabat, Morocco

## Abstract

**Background:**

Several studies have observed an inverse relationship between osteoporosis and spinal osteoarthritis, the latter being considered as possibly delaying the development of osteoporosis. The aim of this study was to determine the association between individual radiographic features of spine degeneration, bone mineral density (BMD) and bone-turn over markers.

**Methods:**

It was a cross sectional study of 277 post menopausal women. BMD of all patients was assessed at the spine and hip using dual-energy X-ray absorptiometry. Lateral spinal radiographs were evaluated for features of disc degeneration. Each vertebral level from L1/2 to L4/5 was assessed for the presence and severity of osteophytes and disc space narrowing (DSN). For Bone turn-over markers, we assessed serum osteocalcin and C-terminal cross-linking telopeptide of type I collagen (CTX). Linear regressions and partial correlation were used respectively to determine the association between each of disc degeneration features, BMD, and both CTX and osteocalcin.

**Results:**

Mean age of patients was 58.7 ± 7.7 years. Eighty four patients (31.2%) were osteoporotic and 88.44% had spine osteoarthritis. At all measured sites, there was an increase in BMD with increasing severity of disc narrowing while there was no association between severity of osteophytes and BMD. After adjustment for age and BMI, there was a significant negative correlation between CTX and DSN. However, no significant correlation was found between CTX and osteophytes and between osteocalcin and both osteophytes or DSN.

**Conclusion:**

In post menopausal women the severity of disc narrowing, but not osteophytes, is associated with a generalized increase in BMD and a decreased rate of bone resorption. These results are consistent with the hypothesis that osteoarthritis, through DSN, has a protective effect against bone loss, mediated by a lower rate of bone resorption. However, spine BMD is not a relevant surrogate marker for the assessment of osteoporosis in the spine in patients with osteoarthritis and debate as to the relationship between OA and OP is still open because of the contradictory data in the literature.

## Background

Osteoporosis (OP) and osteoarthritis (OA) are two common age-related skeletal disorders responsible for major health expenses in the elderly. While OA is a joint disease characterized by degeneration of articular cartilage and bone remodeling that may affect different sites and involve peripheral or axial joints, OP is characterized by low bone mass, and microarchitectural deterioration of bony tissue, with a consequent increase in bone fragility and susceptibility to fractures. According to the WHO criteria, osteoporosis can also be defined as a value of bone mineral density (BMD) more than 2.5 standard deviations below the young normal mean.

It would be anticipated that osteoporosis and osteoarthritis frequently coexist due to their high prevalence in elderly women but the association between these conditions is still controversial [1,2] even after years of research since the first results indicate an apparent inverse relationship [3,4]. Indeed, many studies have shown an association between high bone mineral density at the spine and hip and OA of the hips, knees or hands [5-8]. However, there are conflicting findings in the few published studies on the association between bone mass and degenerative disease in the spine, the latter being characterized by disc space narrowing (DSN) and the presence of vertebral osteophytes. Most [9-13], though not all [14-16] studies that examined the association between osteophytes and bone mass at the spine and distant sites including the hip, suggest that they are linked to an increased bone mass. Results are also discordant about the association between DSN and BMD at distant sites. In a population of patients with OA of the hip, isolated DSN without osteophytes was not associated with high bone mass [17]. In contrast, in a general population, those with isolated DSN have a higher BMD in the spine (but not in the hip) than those without [9].

In order to understand the underlying mechanism of the interaction between bone mass and OA, noninvasive biochemical assays for markers of bone resorption (which the CTX-I is the most specific and sensitive one), and bone formation have been developed and enabled estimation of bone turnover. Few studies of biochemical markers have been reported in subjects with spinal OA, results have also been conflictual [18-20].

We undertook this study to determine the association between radiographic features of lumbar disc degeneration, namely osteophytes and DSN, and BMD at different measured sites, as well as to investigate the underlying mechanism at the tissue level through assessment of biochemical markers of bone metabolism.

## Methods

### Subjects

The study involved 277 consecutive ambulatory postmenopausal women living in urban centre of Morocco and sent to our outpatient Bone Densitometry Center. Recruitment was based on voluntary enrolment. Written informed consent was obtained from all subjects and the study was approved by the Ethical Committee of El Ayachi University Hospital of Rabat-Sale. We excluded from the study all patients with a history of: (1) taking drugs known to influence bone metabolism in the past two years, such as vitamin D, calcium, corticosteroids, bisphosphonates, sodium fluoride, raloxifene, strontium ranelate, teriparatide and hormone replacement therapy; (2) musclo skeletal, thyroid, parathyroid, adrenal, hepatic, or renal disease; (3) malignancy; and (4) hysterectomy.

### Data collection and measurements

Each patient completed a questionnaire on sociodemographic parameters and osteoporosis risk factors such as female sex, age higher than 60 years, family history of osteoporosis, early menopause, low BMI, smoking, sedentary lifestyle, long term (≥3 months) corticosteroid use and excessive alcohol consumption. Weight and height were measured without clothes or shoes at the time of bone densitometry measurements. The body mass index (BMI) was calculated as body weight divided by height squared (Kg/m^2^).

### Bone mineral density (BMD) measurements

Lumbar spine, trochanter, femoral neck and total hip BMD were measured by dual-energy X-ray absorptiometry with a Lunar prodigy densitometer. Only vertebras with scoliosis have been excluded from BMD test. Daily quality control was carried out by measurement of a Lunar phantom. At the time of the study, phantom measurements showed stable results. The in vivo precision error for dual-energy X ray absorptiometry, expressed as coefficient of variation, was 0.9% at the lumbar spine and 1% at the femoral neck. Both T and Z scores were obtained. In the T-score calculations, the manufacturer's ranges for European reference population were used because of the absence of a Moroccan data base.

### Assessment of lumbar spine degeneration

**Figure 1:**
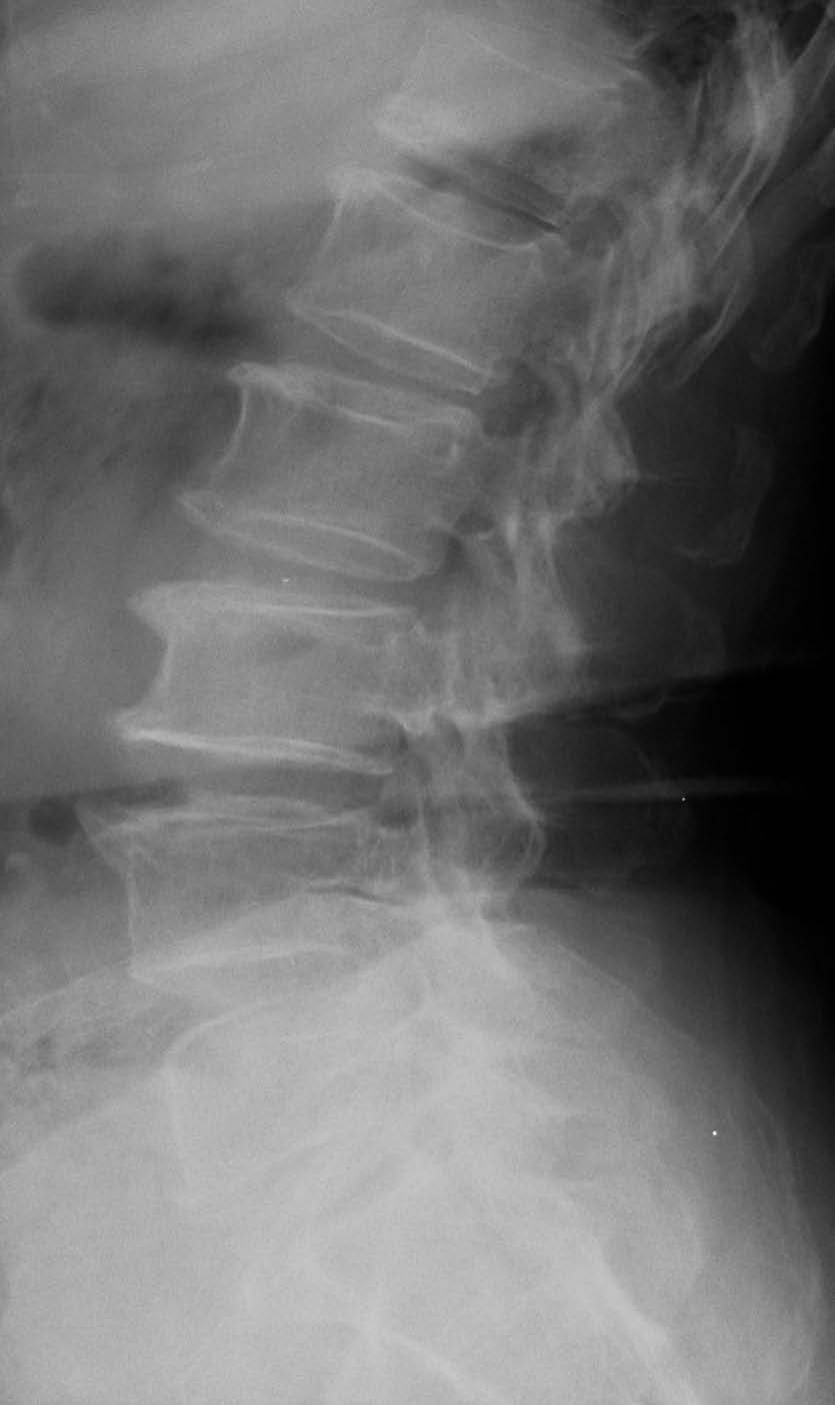
**Osteophytosis and DSN demonstrated on a lateral lumbar spine X-ray**.

Lumbar spine radiographs were taken according to a standard protocol with the film centred at L2 (Figure 1). The radiographs were subsequently evaluated by a single observer for the presence of the individual radiographic features of disc degeneration. Each vertebral level from L1/2 to L4/5 was assessed for the presence and severity of osteophytes and DSN, using a semiquantitative score (grade 0, none; grade 1, mild; grade 2, moderate; grade 3, severe) [21]. We defined, for each radiographic feature, two summary statistics: ''MAX'', which was the grade of the most severely affected vertebral level per subject (from L1/2 to L4/5) and which could range from 0 to 3, and ''SUM'', the sum of the four vertebral specific grades per subject which thus could range from 0 to 12.

### Biochemical measurements

Morning fasting blood was collected from every subject for the measurement of the following parameters: osteocalcin and C-terminal cross-linking telopeptide of type I (CTX). Both parameters were measured by immunochemoluminometric assay (Elecsys, Roche diagnostics, Mannheim, Germany). Intra- and interassay variances were 5% and 7%, and the normal range were 15-46 ng/ml for osteocalcin, and 0.3-0.6 ng/ml for CTX.

### Statistical analysis

Statistical analysis was performed with SPSS for Windows 13.0 (SPSS Inc., Chicago, IL, USA). Population descriptions are expressed as mean ± standard deviation (SD) for continuous variables and as percentage distributions for discrete variables.

Linear regression was used to determine the association between each of the two radiographic features (using both MAX and SUM) and BMD (dependent variable) at spine and femoral sites. Adjustments were made for age and BMI. We examined MAX as a continuous variable to test for any trend of increasing bone mass with increasing grade of radiographic feature. We examined SUM as a continuous variable to test for any trend of increasing bone mass with increasing grade of feature with the results expressed as β coefficients and 95% confidence intervals.

Partial correlation after adjusting for age and BMI was performed to determine the strength of the association between each of the radiographic features (using both MAX and SUM) and CTX and Osteocalcin. p values lower than 0.05 were considered as statistically significant.

## Results

### Clinical characteristics

Characteristics of participants enrolled in this cross sectional study are shown in table 1. Mean age and BMI were 58.7 ± 7.7 years and 29.08 ± 4.35 kg/m^2 ^respectively. 123 women (44.4%) had overweight (BMI > 25) and 101 (36.5%) were obese. 88.44% of the 277 included in the study had spine osteoarthritis and 31.2% were osteoporotic. 43.5% had osteopenia, 46.6% of them had at least a prevalent vertebral fracture and 12.3% had a history of low trauma peripheral fractures. The prevalence of osteophytes and lumbar DSN was 87.5% and 47.2% respectively. Most of the patients (78.8%) had a grade 1 of osteophytes. For DSN, 37.8%, 7.2% and 2.2% of the patients had a grade 1, 2 and 3 respectively. Mean level of CTX I and osteocalcin was 0.49 ± 0.25 and 24.56 ± 13.56 respectively.

**Table 1:** Subject characteristics

Variable	All subjects (N = 277)	
	Mean ± SD	
Age (years)	58.7 ± 7.7	
Age of menopause (years)	47.30 ± 5.28	
Weight (kg)	71.38 ± 11.60	
Height (m)	1,56 ± 0,06	
BMI (Kg/m^2^)	29.08 ± 4.35	
Lumbar spine BMD (g/cm^2^)	0.966 ± 0.159	
Femoral neck BMD (g/cm^2^)	0.848 ± 0.130	
Femoral trochanter BMD (g/cm^2^)	0.693 ± 0.120	
Total femoral BMD (g/cm^2^)	0.892 ± 0.134	
CTX I	0.49 ± 0.25	
Osteocalcin	24.56 ± 13.65	
		
	n	%
Prevalence of osteoporosis	84	31,2
Prevalence of spine osteoarthritis	245	88.44

BMD: bone mineral density

BMI: body mass index

DSN: disc space narrowing

CTX: C-terminal cross-linking telopeptide of type I collagen

### Maximum grade of radiographic feature (MAX) and BMD

#### ✓ Lumbar spine BMD

The association between BMD at the lumbar spine and the maximum grade of each radiographic feature per subject (MAX) is shown in table 2. After adjusting for age and BMI, lumbar spine BMD increased with increasing grade of disc space narrowing. For example, the mean age-adjusted lumbar spine BMD rises from 0.95 g/cm^2 ^for patients without DSN to 1.118 g/cm^2 ^for patients with grade 3 of DSN (p = 0.001). This trend of increasing BMD with increasing grade of DSN persisted after adjusting for BMI. However, there was no association between lumbar spine BMD and osteophytes.

**Table 2:** Maximum grade (MAX) of individual radiographic features and BMD at different measured sites in 277 postmenopausal women

			Lumbar spine BMD		Trochanter BMD		Femoral neck BMD		Femoral total BMD	
	**n**	**(%)**	**Mean (SD)**	**Multivariate analysis β (95% CI)**	**Mean (SD)**	**Multivariate analysis β (95% CI)**	**Mean (SD)**	**Multivariate analysis β (95% CI)**	**Mean (SD)**	**Multivariate analysis β (95% CI)**

**Osteophytes**										
Grade 0	34	(12.5)	0.983 (0.187)	1	0.710 (0.125)	1	0.884 (0.125)	1	0.925 (0.149)	1
Grade 1	219	(78.8)	0.963 (0.153)	-0.01 (-0.05 to 0.05)	0.693 (0.121)	-0.01 (-0.04 to 0.03)	0.845 (0.134)	-0.02 (-0.07 to 0.02)	0.889 (0.132)	-0.01 (-0.01 to 0.02)
Grade 2	11	(4.0)	0.937 (0.217)	0.02 (-0.07 to 0.13)	0.643 (0.103)	-0.01 (-0.08 to 0.06)	0.822 (0.884)	-0.01 (-0.08 to 0.08)	0.849 (0.110)	0.01 (-0.07 to 0.08)
Grade 3	13	(4.7)	0.989 (0.140)	-0.01 (-0.08 to 0.06)	0.693 (0.120)	-0.01 (-0.08 to 0.06)	0.831 (0.992)	-0.03 (-0.11 to 0.04)	0.891 (0.128)	-0.01 (-0.08 to 0.06)
										
**DSN**										
Grade 0	146	(52.8)	0.953 (0.156)	1	0.686 (0.121)	1	0.845 (0.144)	1	0.896 (0.137)	1
Grade 1	105	(37.8)	0.974 (0.255)	0.02 (-0.01 to 0.06)	0.674 (0.114)	0.01 (-0.01 to 0.04)	0.849 (0.110)	0.01 (-0.02 to 0.04)	0.885 (0.130)	-0.01 (-0.03 to 0.02)
Grade 2	20	(7.2)	0.966 (0.232)	0.07 (-0.01 to 0.14)*	0.689 (0.152)	0.05 (-0.01 to 0.10)*	0.870 (0.137)	0.08 (0.02 to 0.14)*	0.893 (0.146)	0.06 (0.01 to 0.12)*
Grade 3	6	(2.2)	1.118 (0.861)	0.19 (0.07 to 0.30)*	0.683 (0.120)	0.01 (-0.06 to 0.10)	0.843 (0.931)	0.02 (-0.07 to 0.12)	0.909 (0.107)	0.05 (-0.04 to 0.14)

*p < 0.05

Adjustment for age and BMI in the multivariate analysis

BMD: bone mineral density

#### ✓ Femoral BMD

The association between the MAX of each radiographic features and BMD at the neck, tochanter, and total femoral is shown in table 2. After adjusting for age, patients with grade 2 of DSN had an increased BMD at all measured sites than other patients. For example, the mean age-adjusted femoral neck BMD was 0.845 g/cm^2 ^for patients without DSN and 0.870 g/cm^2 ^for patients with grade 2 of DSN (p = 0.003). This trend of increasing BMD in patients with grade 2 of DSN persisted after adjusting for BMI. In contrast, there was no association between femoral BMD and osteophytes.

### Summary score for radiographic features (SUM) and BMD

The influence of the radiographic features as assessed using the total score (SUM) across all the four intervertebral levels, on BMD at all measured sites, is shown in table 3. Results are expressed as β coefficients which may be interpreted as the absolute change in BMD (g/cm^2^) per unit change in score. BMD at all measured sites increased with SUM DSN. For example, after age adjustment, lumbar BMD increased by a value of 0.02 g/cm^2 ^for each unit change in the total DSN score. The observed associations remain unchanged after adjustment for age and BMI. However, there was no association between BMD at all measured sites and SUM osteophytes.

**Table 3:** Total scores (SUM) of individual radiographic features and BMD at different measured sites, after adjusting for age and BMI, in 277 postmenopausal women

	Lumb spine BMD β (95% CI)	Trochanter BMD β (95% CI)	Femoral neck BMD β (95% CI)	Total femoral BMD β (95% CI)
**Osteophyte**	-0.01 (-0.01 to 0.01)	-0.01 (-0.01 to 0.01)	-0.01 (-0.01 to 0.01)	-0.01 (-0.01 to 0.01)
**DSN**	0.02 (0.01 to 0.03)*	0.01 (0.01 to 0.02)*	0.01 (0.01 to 0.02)*	0.01 (0.01 to 0.02)*

*p < 0.05

BMD: bone mineral density

DSN: disc space narrowing

CI: confidence interval

### Relationship between osteoarthritis and bone turn over markers

A significant decrease in CTX-I levels associated with lumbar spine disc degeneration was observed (table 4). Indeed, after adjustment for age and BMI there was a significant negative correlation between CTX and MAX DSN (r adjusted = -0.192, p = 0. 026). The level of CTX was also negatively associated with SUM DSN (r adjusted = -0.209, p = 0.019). However, no significant correlation was found between CTX and MAX or SUM osteophytes and between osteocalcin; and both SUM and MAX DSN or osteophytes.

**Table 4:** Partial Correlation showing a significant negative correlation between CTX and DSN in 277 postmenopausal women

	CTX r adjusted	Osteocalcin r adjusted
**MAX osteophyte**	-0.097	-0.032
**SUM osteophyte**	-0.137	-0.135
**MAX DSN**	-0.192*	-0.036
**SUM DSN**	-0.209*	-0.075

*p < 0.05

Adjustement for age and BMI

CTX: C-terminal cross-linking telopeptide of type I collagen

DSN: disc space narrowing

## Discussion

Our data show that in post menopausal women increasing severity of disc space narrowing, but not osteophytes, is related to increasing bone mineral density at all measured sites. The severity of disc space narrowing was also associated to a decrease in bone resorption, without any effect on bone formation.

Marked differences in the prevalence of spinal degeneration features occur in association with older age, female sex, post menopausal women and obesity. In our study, the prevalence of spine osteoarthritis was high (88.44%). 78.7% and 47.7% of patients had at least one osteophyte or at least one DSN respectively. This can be explained by age (Mean (SD) age was 58.7 ± 7.7), sex, overweight (44.4% had BMI > 25) and obesity (36.5% of the patients). Mean (SD) BMI was 29.08 ± 4.35.

On the other hand, several studies observe an inverse relationship between OP and spine OA, the latter being considered as possibly delaying the development of OP [10,18,19,21]. Our data showed that increasing BMD in spine OA is more related to DSN than to osteophytes. Indeed, after adjusting for age and BMI, no association was found between BMD at all measured sites and the severity of osteophytes. This can be explained by the fact that the majority of the patients (78.8%) had only a grade 1 of osteophytes which corresponds to a mild involvement of this radiographic feature. The influence of osteophytes on BMD has been the focus of various studies, which showed, in contrast to our findings [6,10,12,16-18], that spinal BMD was greater in vertebrae with osteophytes. Several other studies have examined the association between osteophytes and bone mass at distant sites including the hip and most [6,9-13,18], though not all [14-16] suggest that they are also linked to an increased bone mass. However, it is important to note that spine disc degeneration can hinder the interpretation of spine BMD: osteophytes cannot be distinguished from vertebral bone mineral using BMD area measurements and may even in some cases overestimate the measurements of bone mass in the affected areas. Therefore, it has been suggested that spine BMD is not a relevant surrogate marker for the assessment of osteoporosis in the spine in patients with osteoarthritis [9,12].

Relatively little however is known about the association between bone mass and DSN and there are also conflicting findings in the few published studies on the subject [12-14,16,17]. Thus, in a population of patients with OA of the hip, isolated DSN without osteophytes was not associated with high bone mass [17]. In contrast, Pye et al showed an association between DSN and increasing BMD at the spine but not at the hip [9]. Our data show that increasing severity of DSN is associated with increasing BMD at all measured sites and support, through the DSN results, the hypothesis that degenerative disc disease is inversely linked with osteoporosis.

The mechanism is unknown though several are possible; including confounding by environmental or constitutional factors, hormonal, metabolic, and genetic factors [22,23]. Our results remained unchanged after adjustment for age and BMI, suggesting that they do not play a major role in explaining the observed associations.

Few studies of biochemical markers have been reported in subjects with spinal OA, with discordant results [18-20]. Peel et al [18] and El Miedany et al [19] have shown that spinal OA is associated with a generalized increase in BMD and decreased levels of serum and urinary biochemical markers of bone formation and bone resorption in patients with spine disc degeneration. It has been suggested that the protective effect of spinal OA against OP may be mediated through decreased rate of bone turnover. Garnero et al [20] in a large cohort of untreated postmenopausal women participating in the OFELY prospective study have shown recently that lumbar spine DSN, but not osteophytes, is strongly associated with increased CTX-II degradation, independently of age and BMI. Our results agree partially with what has been reported by Peel et al. and El Miedany et al [18,19] who found in their studies a decrease in bone resorption markers in women with spinal OA. However, we did not find any effect of spine disc degeneration on bone formation. Moreover, no association was found between the severity of osteophytes and either increasing BMD or CTX levels.

Our study is limited by its cross-sectional design and the use of a semiquantitative score to classify the radiographic features of disc degeneration. As with any subjective evaluation, this is subject to errors of interpretation which may result in misclassification. However, this defect applies to all studies in this field because the same grading systems are used universally. Moreover, only 9.4% of our subjects had disc space narrowing that was associated with a significantly increased bone mineral density and further studies on a larger population are necessary to confirm our findings. Finally, and as mentioned above, spine BMD is not a relevant surrogate marker for the assessment of osteoporosis in the spine in patients with osteoarthritis. Indeed, measurements of BMD taken by DXA are the most accurate procedure for the diagnosis of osteoporosis nowadays. However, these measurements are two-dimensional and when made with an anterior-posterior projection, the most used incidence, this procedure has the disadvantage of measuring the density of all the mineral components encountered in the X-ray pathway, including osteophytes, bone sclerosis, disk space narrowing, spondylolisthesis, vertebral fractures, and vascular and extra-vertebral calcifications. Moreover, some studies has actually shown that obesity and these alterations can influence bone mineral density results. Although this defect applies to all studies in this field because DXA is used universally, it would be interesting to study OA in other places.

## Conclusion

This study showed that in post menopausal women the severity of disc narrowing, but not osteophytes, was associated with a generalized increase in BMD and a decreased rate of bone resorption. These results are consistent with the hypothesis that osteoarthritis, through disc space narrowing, has a protective effect against bone loss, mediated by a lower rate of bone resorption. However, spine BMD is not a relevant surrogate marker for the assessment of osteoporosis in the spine in patients with osteoarthritis and debate as to the relationship between OA and OP is still open because of the contradictory data in the literature.

## Competing interests

The authors declare that they have no competing interests.

## Authors' contributions

LI participated in study design and drafted the manuscript. FA conceived the original idea for the study, supervised its design, performed the statistical analysis and gave critical comments on the draft manuscript. SR enrolled patients, participated in data acquisition and critical revision of the manuscript. LB enrolled patients, participated in data acquisition and critical revision of the manuscript. IH enrolled patients, participated in data acquisition and critical revision of the manuscript. FZA enrolled patients, participated in data acquisition and critical revision of the manuscript. HK enrolled patients, participated in data acquisition and critical revision of the manuscript. LE enrolled patients, participated in data acquisition and critical revision of the manuscript. RA conceived the study and performed the statistical analysis. NHH participated in the study design, coordinated the study and gave critical comments on the draft manuscript. All authors read and approved the final manuscript.

## Pre-publication history

The pre-publication history for this paper can be accessed here:

http://www.biomedcentral.com/1472-6874/10/25/prepub
